# Automated Cardiac Reorientation and Slice Extraction in Cine Coronary CT Angiography: Agreement with Cardiovascular Magnetic Resonance

**DOI:** 10.3390/tomography12080114

**Published:** 2026-08-14

**Authors:** An-Yu Sun, Li-Yueh Hsu, Andrew Heller, Matthew Jacobs, W. Patricia Bandettini, Lin-Ching Chang, Marcus Y. Chen

**Affiliations:** 1 National Heart, Lung, and Blood Institute, National Institutes of Health, Bethesda, MD 20892, USA; 2 Department of Computer Science, Catholic University of America, Washington, DC 20064, USA

**Keywords:** cine coronary CT angiography, cardiac magnetic resonance, cardiac coordinate system, long-axis plane, short-axis plane, automated segmentation, automated reorientation

## Abstract

Visualization of coronary computed tomography angiography (CCTA) in standard cardiac planes requires reorientation of the three-dimensional image volume because the heart is double-oblique relative to the axial scan plane. To standardize this process across multi-phase cine CCTA, we developed an automated framework that segments cardiac structures and detects anatomic landmarks to define a patient-specific cardiac coordinate system. Landmark-derived left ventricular (LV) long-axis and LV-to-right ventricular transverse vectors were used to compute a rotation matrix for reorienting all 20 cine CCTA volumes. Six fixed slice planes were then defined to extract three long-axis (LAX) and three short-axis (SAX) slices at each phase. Matched CCTA and cardiac magnetic resonance (MR) slices were compared in 25 patients at diastasis, end-diastole (ED), and end-systole (ES), using automated segmentation-derived measurements of the combined LV cavity and LV myocardium area (LV + LVM). Agreement was evaluated with intraclass correlation coefficients (ICCs), Bland–Altman analysis, and repeated-measures mixed-effects models. ICCs were 0.927 (95% CI, 0.895 to 0.950) at diastasis, 0.950 (95% CI, 0.928 to 0.966) at ED, and 0.919 (95% CI, 0.887 to 0.943) at ES. Bland–Altman analysis showed small CT-positive biases of 1.05, 0.47, and 1.86 cm^2^ at diastasis, ED, and ES, corresponding to relative biases of 2.8%, 1.2%, and 5.8% of the phase-specific mean MR LV + LVM area. Mixed-effects models showed no statistically significant phase-level bias at diastasis or ED, whereas ES showed a small but significant CT-positive bias. These findings support automated CCTA reorientation as an interpretable framework for reproducible LAX/SAX slice extraction and future quantitative functional cine CCTA analysis.

## Simple Summary

Cardiac long-axis and short-axis views in coronary CT angiography (CCTA) require reorientation of the 3D image volume because the heart is double-oblique relative to the axial scan plane. This study presents an automated cine CCTA framework that uses segmentation-derived landmarks to define a patient-specific cardiac coordinate system, reorient 3D volumes, and extract reproducible long-axis and short-axis slices for comparison of left ventricular area measurements with cardiac MR. The method showed strong agreement between CT- and MR-derived measurements across cardiac phases, with the strongest agreement and smallest bias at end-diastole, and small CT-positive biases overall.

## 1. Introduction

Visualization of cardiac anatomy in two-dimensional (2D) multiplanar reformatted long-axis (LAX) and short-axis (SAX) views requires reorientation of the three-dimensional (3D) image volume because the heart is double-oblique relative to the axial scan plane. The American Heart Association (AHA) recommends that cardiac imaging modalities define, orient, and display the heart using the left ventricular (LV) long axis, commonly defined as the line connecting the center of the mitral valve plane and the LV apex, with selected planes oriented 90° relative to that axis [1]. In coronary computed tomography angiography (CCTA), this reorientation is particularly important because routine axial acquisition does not directly provide these standard cardiac views. Recent CCTA auto-localization work has also emphasized LV long-axis localization as a key post-processing step for consistent cardiac orientation and quantitative analysis [2].

In routine CCTA workflows, LAX and SAX views are usually generated manually with multiplanar reformation. Although this workflow is common in clinical practice, it requires operator expertise and may introduce interobserver variability. In time-resolved cine CCTA, manual plane relocalization across cardiac phases can amplify small orientation differences and create apparent changes caused by inconsistent reformatting rather than true cardiac motion. Consistent LAX and SAX geometry is also clinically relevant for AHA-style segmental localization, regional wall-motion review, and reproducible serial functional assessment [1].

Cardiac magnetic resonance (MR) is commonly used as a reference modality for cardiac chamber assessment, but MR plane prescription is also operator-dependent. Society for Cardiovascular Magnetic Resonance (SCMR) guidance describes sequential scout-based planning of the LV two-chamber, three-chamber, and four-chamber LAX views, followed by SAX cine planning using anatomic landmarks [3]. SCMR post-processing guidance also emphasizes annular-plane correction and reader review of automated analysis results [4]. Together, these recommendations indicate that cardiac MR visualization, downstream analysis, and measurements may be influenced by localizer quality, operator-dependent landmark and phase selection, breath-hold position, annular-plane correction, and automated analysis quality.

An automated coordinate system anchored to segmented cardiac anatomy could reduce variability in LAX and SAX plane selection for cardiac CT and provide a consistent geometric basis for AHA segmental assignment, regional wall-motion review, downstream processing, and quantitative cine analysis. Prior CCTA work validated automatic LV long-axis localization in single-phase CCTA volumes by comparing computer-derived axes with expert-defined long-axis references [2]. In this study, we extend this concept to multi-phase cine CCTA by presenting an automated framework that reorients 3D cardiac volumes, extracts LAX and SAX slices across cardiac phases, and compares the resulting combined LV cavity and LV myocardium area measurements with those from cardiac MR. Our goal was to evaluate the method using cross-modality measurement agreement rather than rotation-angle agreement alone.

## 2. Materials and Methods

All examinations were approved by the Institutional Review Board at the National Institutes of Health, and all participants provided written informed consent. This retrospective technical evaluation study included a nonconsecutive sample of 25 patients examined between 2015 and 2020. Inclusion required multiphase cine CCTA and cardiac MR performed within 24 h of each other. Patients were excluded if CCTA images contained artifacts from external metallic objects or wires, or if suboptimal cardiac MR LAX prescription precluded reliable CT–MR slice matching.

### 2.1. Image Acquisition

CCTA was performed on a 320-detector-row scanner (Aquilion ONE Genesis, Canon Medical Systems, Tochigi, Japan). Iodinated contrast was administered, and prospective ECG-triggered acquisition began when attenuation in the descending aorta reached a prespecified threshold. Images were reconstructed with a 512 × 512 matrix, 0.26–0.43 mm pixel spacing, and 0.25–0.5 mm slice thickness. Cine CCTA images were reconstructed at 5% increments from 0% to 95% R–R of the cardiac cycle, yielding 20 3D volumes per patient. All CCTA volumes were resampled to 1.0 mm isotropic voxels for segmentation, reorientation, slice extraction, and measurements.

Cardiac MR was performed on a 3.0 T scanner (Skyra, Siemens Healthineers, Erlangen, Germany) using an ECG-gated steady-state free precession sequence. Cine cardiac MR images were reconstructed at approximately 3.3% increments across the cardiac cycle, yielding 30 phases per patient. An experienced technologist first acquired single-shot localizers and used them to prescribe an initial scout cine series. This dynamic scout series was used to visualize cardiac motion, identify diastasis, and refine plane placement. Three LAX cine slices were then acquired, followed by a SAX cine stack from base to apex.

### 2.2. Automated 3D Cardiac Reorientation

A fully automated framework was developed to reorient cine CCTA volumes and extract reproducible LAX and SAX slices. First, our previously developed DeepHeartCT convolutional neural network (CNN)-based segmentation framework [5] was applied to the phase-15 CCTA volume, which corresponded to approximately 75% of the cardiac cycle and served as the diastatic reference phase. The CNN was trained for multi-structure CCTA segmentation using labels generated by a hybrid multi-atlas segmentation framework [6,7] and was used to label the LV cavity, left atrium (LA), right atrium, right ventricle (RV), ascending aorta, and LV myocardium. These labels were used for subsequent anatomic landmark detection and slice extraction.

**Figure 1:**
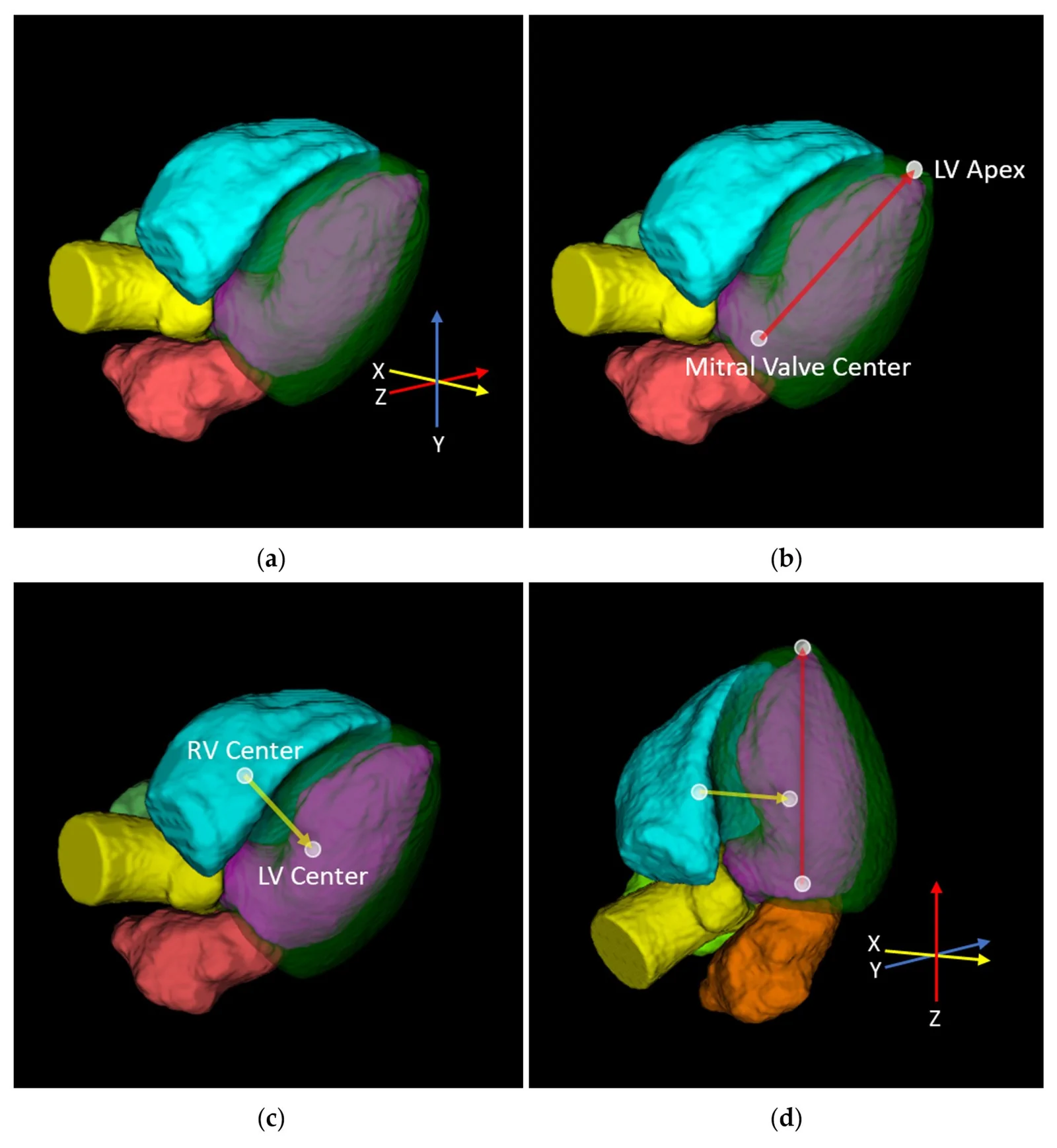
Automated cardiac reorientation using segmentation-derived landmarks. (**a**) Original 3D volume before reorientation, with target coordinate axes shown in the lower right. (**b**) The LV long-axis vector, shown in red, connects the mitral valve center and LV apex for *z*-axis alignment. (**c**) The LV and right ventricular (RV) centers define the LV-RV transverse vector, shown in yellow, for *x*-axis alignment. (**d**) Reoriented 3D volume after applying the 3D rotation matrix, with the LV long-axis and LV-RV transverse vectors aligned with the target coordinate axes. Colors indicate structures: orange, left atrium; magenta, left ventricle; green, LV myocardium; cyan, right ventricle; lime, right atrium; yellow, ascending aorta.

The framework defined 3D cardiac orientation using segmentation-derived landmarks (Figure 1). The mitral valve center was defined as the centroid of the voxel overlap between the LA and LV cavity masks after each mask was morphologically dilated by 3 mm. The aortic valve center, subsequently used to define LAX_3, was defined analogously from the LV cavity and ascending aorta masks. The LV apex was identified as the segmented LV surface point farthest from the mitral valve center. The LV long-axis vector was then defined from the mitral valve center to the LV apex. The LV and RV centers were defined as the centroids of their respective cavity masks.

Reorientation parameters were estimated from two segmentation-derived anatomic vectors. First, the LV long-axis vector was used to estimate the rotation angle required to align the mitral valve-to-apex direction with the image *z*-axis, establishing longitudinal orientation. In the aligned coordinate frame, the LV–RV transverse vector was defined as the 3D vector from the LV centroid to the RV centroid. A second rotation angle around the image *z*-axis was estimated to align this vector with the image *x*-axis. A 3D rigid rotation matrix was then computed from these rotations and applied to the CCTA volume, completing 3D cardiac reorientation (Figure 1). Linear interpolation was used for all rotation and resampling operations.

The phase-15 derived rotation matrix, slice-plane orientations, and positions were applied to all 20 cine CCTA phases as a deliberate reproducibility choice. This fixed-plane approach mirrors cardiac MR cine prescription, in which LAX and SAX planes are prescribed from a reference planning phase and then used throughout the cine acquisition. This approach also avoids potential phase-to-phase variation in cardiac-axis orientation and slice position that could occur if cardiac axes were re-estimated and slices were extracted independently at each CCTA phase.

### 2.3. Extraction of Long-Axis and Short-Axis Slices

**Figure 2:**
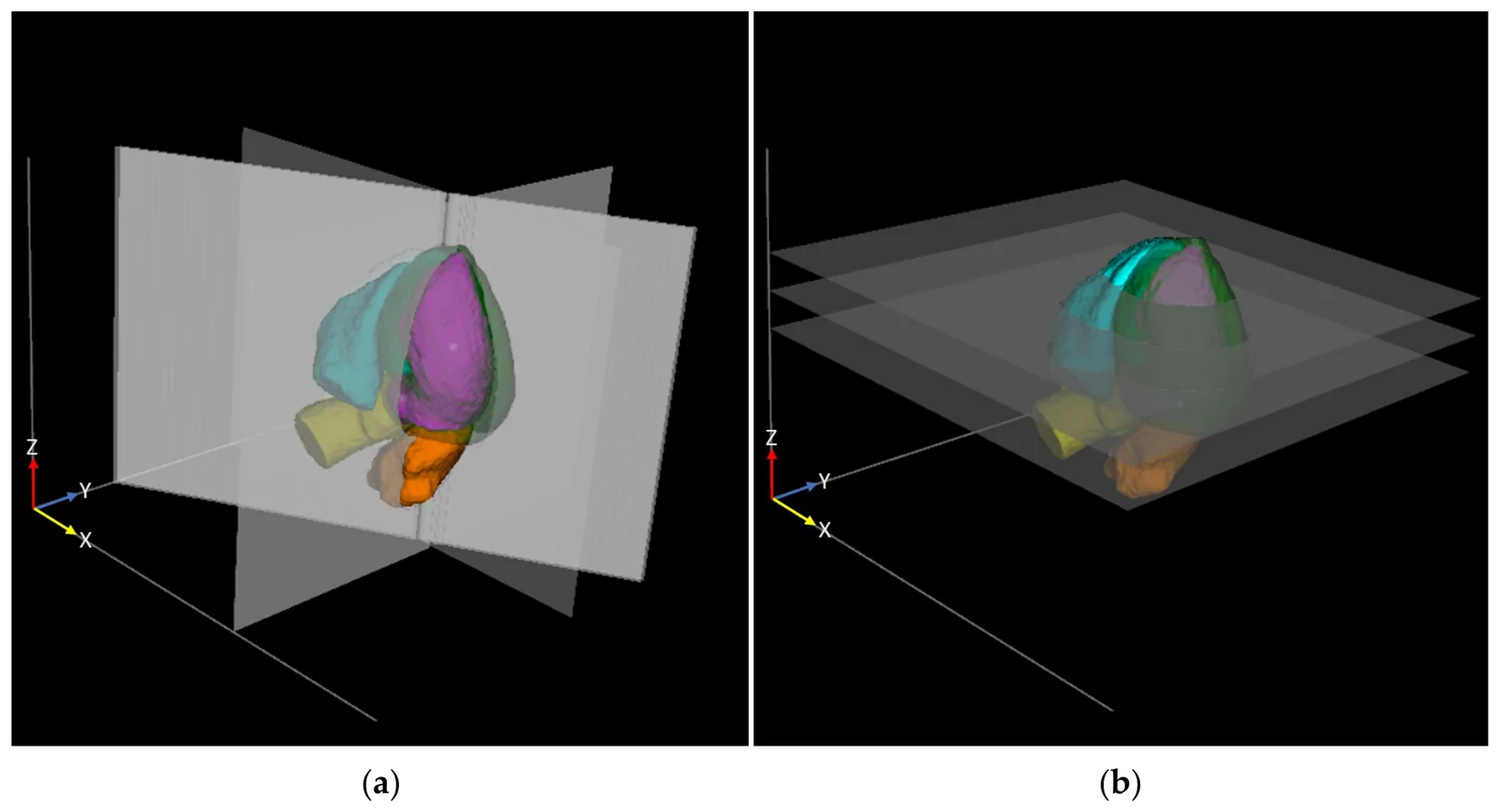
Automated LAX and SAX slice extraction after cardiac reorientation. (**a**) LAX slice planes for the two-chamber (LAX_2), three-chamber (LAX_3), and four-chamber (LAX_4) views. (**b**) SAX slice planes placed at 25%, 50%, and 75% of the LV long-axis distance from the mitral valve center to the LV apex, corresponding to basal (SAX_B), mid-cavity (SAX_M), and apical (SAX_A) sampling slices. Colors indicate structures: orange, left atrium; magenta, left ventricle; green, LV myocardium; cyan, right ventricle; lime, right atrium; yellow, ascending aorta.

After spatial reorientation, LAX and SAX slice planes were computed from the phase-15 cardiac coordinate system. The LAX planes were defined as longitudinal planes parallel to the reoriented image *z*-axis and passing through the LV center. The two-chamber view (LAX_2) was generated from a plane passing through the LV center and parallel to the image *y*-axis. The four-chamber view (LAX_4) was generated from a plane passing through the LV center and parallel to the image *x*-axis. The three-chamber view (LAX_3) was generated from a plane passing through the LV center and the aortic valve center (Figure 2a).

Three representative SAX slices were defined at basal (SAX_B), mid-cavity (SAX_M), and apical (SAX_A) locations by placing slice planes perpendicular to the LV long axis at 25%, 50%, and 75% of the LV long-axis distance from the mitral valve center to the LV apex (Figure 2b). These SAX slices served as reproducible sampling locations and may be further optimized for specific clinical applications. Each LAX and SAX slice was reconstructed as the unweighted arithmetic mean of seven parallel 1 mm-thick slices at offsets of −3, −2, −1, 0, +1, +2, and +3 mm from the prescribed plane.

The same LAX and SAX slice-plane geometry was then applied to all 20 cine CCTA phases to extract the corresponding slices. Runtime was measured on a workstation with an Intel Core i9-10980XE 3.00 GHz CPU and 128 GB RAM. Custom image processing was implemented in Python (version 3.8), and 3D visualization was performed with ITK-SNAP [8].

### 2.4. Cardiac MR Reference Slice and Phase Matching

For SAX slice matching, one basal, one mid-cavity, and one apical slice were manually selected from the full cardiac MR SAX stack to best approximate the 25%, 50%, and 75% locations along the LV long axis used in CCTA. The comparison intentionally avoided the most basal and most apical slices, because the most basal slices may show incomplete LV cavity or myocardial coverage due to LV outflow-tract inclusion and phase-dependent basal motion, particularly between end-diastole and end-systole, whereas the most apical slices are more susceptible to non-axial contraction and small through-plane localization differences.

Phase matching was performed at three time points: diastasis, end-diastole (ED), and end-systole (ES). Diastasis was set at approximately 75% of the cardiac cycle, corresponding to phase-15 of the 20-phase CCTA series and phase 22 of the 30-phase MR cine series. ED was defined as the first reconstructed phase in both modalities, whereas ES was identified as the phase with the smallest LV size in each modality. Mean ES timings was approximately 42% and 40% of the cardiac cycle for CCTA and cardiac MR, respectively.

### 2.5. CT-MR Area Measurements

Agreement between CCTA and cardiac MR was assessed using paired, plane-specific measurements of the combined in-plane area of the LV cavity and LV myocardium (LV + LVM) on each slice. The combined LV + LVM area was selected because it represents the full LV cross-sectional envelope and therefore emphasizes plane-placement agreement while reducing sensitivity to variability in the endocardial blood-pool boundary.

For CT images, LV + LVM area was calculated using the automated DeepHeartCT segmentation framework [5]. For MR images, LV + LVM area was measured in SuiteHeart software (version 5.1.3, NeoSoft, Pewaukee, WI, USA) with automated segmentation followed by manual review and quality assurance.

For each patient, three LAX and three SAX measurements were compared at each of three cardiac phases: diastasis, ED, and ES. Each cardiac phase included 150 paired measurements, comprising six slice-specific measurements per patient. Thus, each patient contributed 18 paired CT-MR measurements, for a total of 450 paired measurements.

### 2.6. Statistical Analysis

Agreement between CT- and MR-derived area estimates was summarized using intraclass correlation coefficients (ICCs). ICCs were calculated using a two-way mixed-effects, single-measure model for absolute agreement and are reported with 95% confidence intervals (CIs) from patient-level bootstrap resampling. Bland–Altman analyses were performed using CT-minus-MR differences, with limits of agreement (LOAs) calculated as the mean bias ± 1.96 times the standard deviation of the differences.

Linear mixed-effects models included a patient-level random intercept to account for repeated measurements within each patient. CT-minus-MR biases from mixed-effects models are reported with 95% CIs and *p* values. All analyses were performed in Python. A two-sided *p* value < 0.05 was considered statistically significant.

## 3. Results

**Figure 3:**
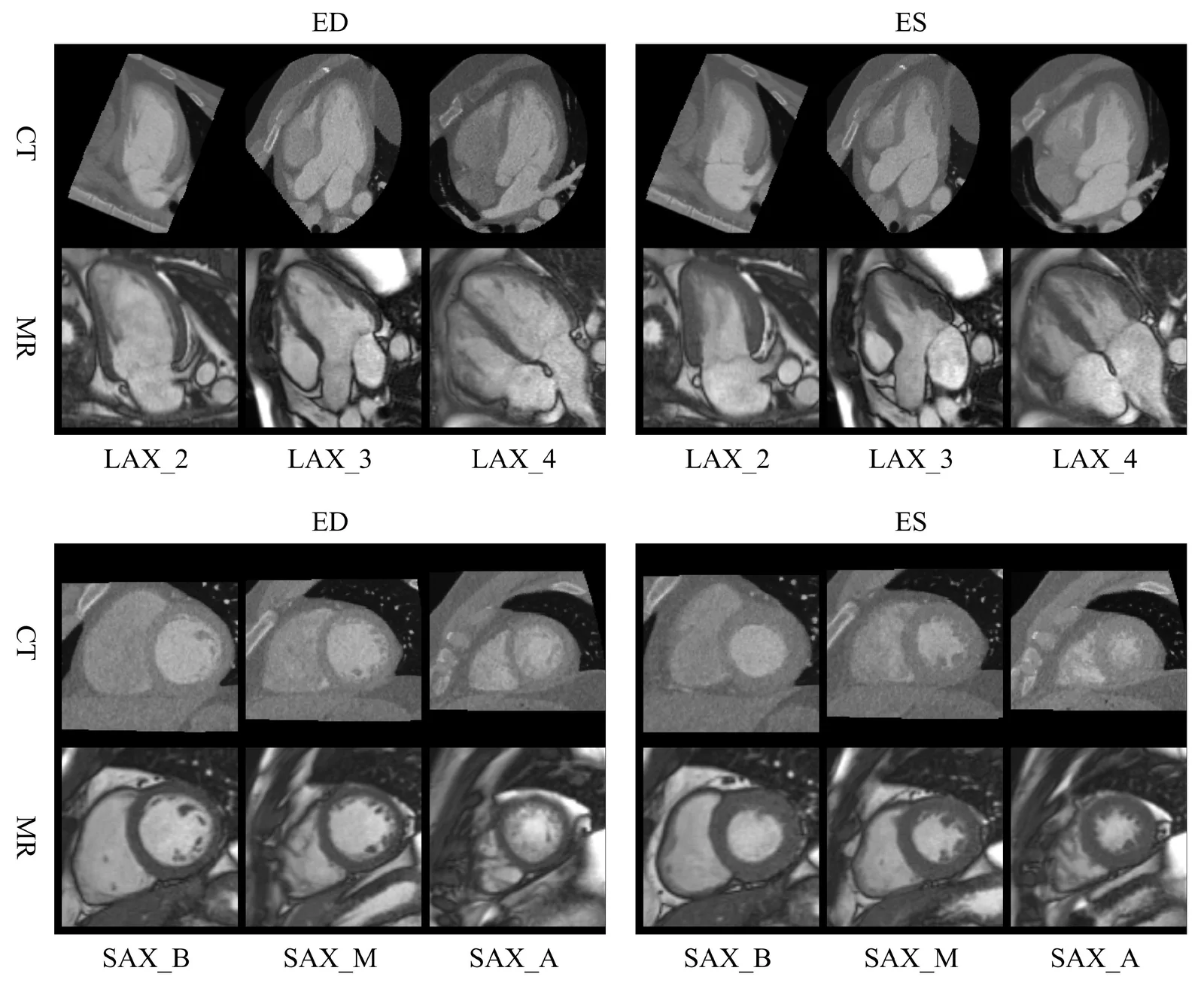
Representative automatically extracted LAX and SAX slices from CCTA and corresponding manually prescribed cardiac MR slices. **Top**: LAX two-chamber (LAX_2), three-chamber (LAX_3), and four-chamber (LAX_4) views at end-diastole (ED) and end-systole (ES). **Bottom**: SAX basal (SAX_B), mid-cavity (SAX_M), and apical (SAX_A) slices at ED and ES.

The clinical characteristics of the study cohort are summarized in Table 1. The processing workflow, including segmentation, volume reorientation, and extraction of the six prescribed 2D slices from all 20 cine volumes, averaged 134 s per cine CCTA case. Representative CT-MR slice matching examples are shown in Figure 3, demonstrating close visual correspondence between automatically extracted CCTA LAX and SAX slices and manually prescribed cardiac MR slices.

Agreement was strong across phases (Table 2). Single-measure absolute-agreement ICCs were 0.927 (95% CI, 0.895 to 0.950) at diastasis, 0.950 (95% CI, 0.928 to 0.966) at ED, and 0.919 (95% CI, 0.887 to 0.943) at ES, with the strongest agreement at ED.

**Figure 4:**
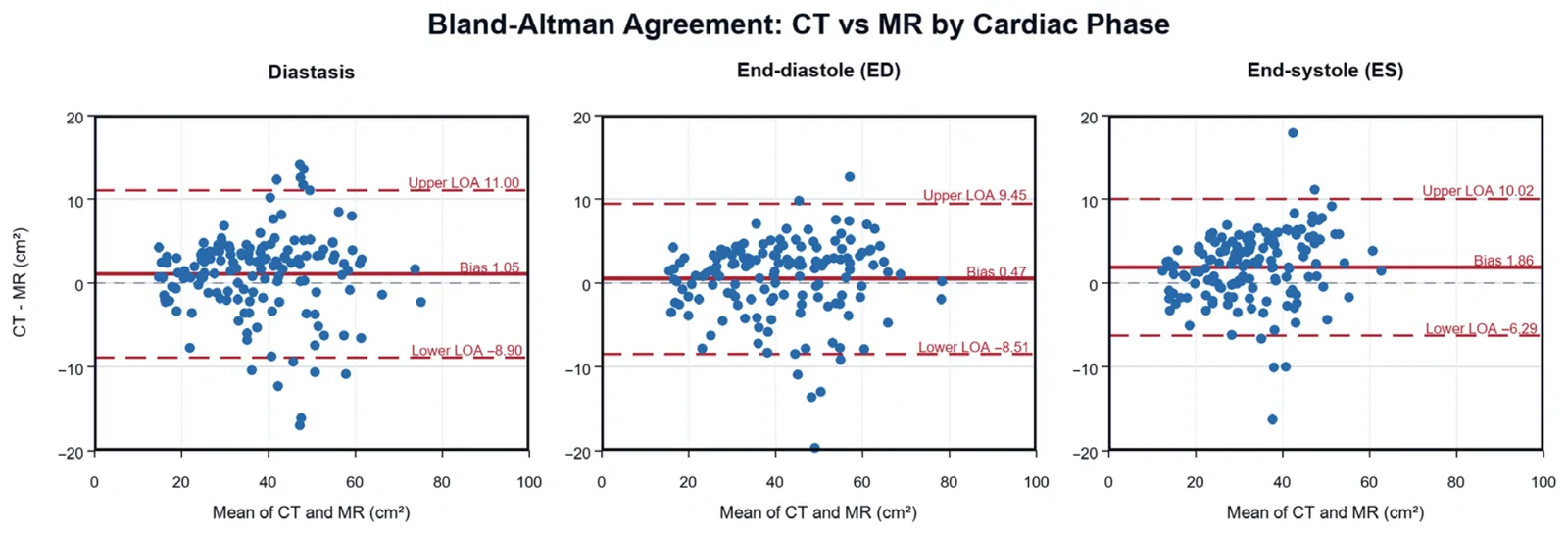
Bland–Altman plots comparing CCTA and cardiac MR LV + LVM area measurements at diastasis, end-diastole, and end-systole. Differences are CT minus MR; positive values indicate larger CT measurements. Horizontal lines indicate mean bias and 95% limits of agreement.

Bland–Altman analyses showed small CT-positive biases, indicating that LV + LVM area measurements were generally larger on CT than on MR (Figure 4). Mean LV + LVM areas on CT and MR were 38.42 and 37.37 cm^2^ at diastasis, 41.26 and 40.78 cm^2^ at ED, and 33.74 and 31.88 cm^2^ at ES. Corresponding biases were 1.05 cm^2^ (95% LOA, −8.90 to 11.00), 0.47 cm^2^ (−8.51 to 9.45), and 1.86 cm^2^ (−6.29 to 10.02) at diastasis, ED, and ES, respectively, representing 2.8%, 1.2%, and 5.8% of the MR-derived phase-specific mean LV + LVM area.

In repeated-measures mixed-effects models (Table 2), CT-minus-MR bias was not statistically significant at diastasis (1.05 cm^2^; 95% CI, −0.11 to 2.21; *p* = 0.076) or ED (0.47 cm^2^; 95% CI, −0.58 to 1.52; *p* = 0.376), whereas ES showed a small but statistically significant CT-positive bias (1.86 cm^2^; 95% CI, 0.93 to 2.80; *p* < 0.001).

Bias varied by LAX and SAX slice location (Table 3). Slice-specific mean CT-minus-MR biases ranged from −4.59 to 5.10 cm^2^ at diastasis, −4.86 to 3.35 cm^2^ at ED, and −2.16 to 4.62 cm^2^ at ES. LAX_3 showed negative bias in all phases (−4.59, −4.86, and −2.16 cm^2^), whereas LAX_2 showed the largest positive bias.

## 4. Discussion

This technical validation study shows that an automated, anatomy-guided CCTA reorientation framework can generate reproducible LAX and SAX slices across cine phases, with LV + LVM area measurements that agree strongly with those from manually prescribed cardiac MR slices. Agreement was strongest at ED, which had the highest ICC and the smallest CT-positive bias. Bland–Altman and mixed-effects analyses showed small CT-positive biases overall. Diastasis and ED showed no statistically significant phase-level bias in the mixed-effects models, whereas ES showed a small but statistically significant CT-positive bias. These results support automated plane prescription as a reproducible foundation for quantitative cine CCTA workflows, consistent with AHA and Society of Cardiovascular Computed Tomography (SCCT) recommendations for cardiac-specific CCTA post-processing and review [1,9].

The repeated-measures analysis accounted for clustering of phase- and slice-specific measurements within each patient. Residual disagreement appeared to be related primarily to differences among slice locations rather than consistent patient-specific offsets. LAX_3 was the only slice with a negative mean CT-minus-MR bias across all phases, whereas LAX_2 showed the largest positive bias; however, both biases were small relative to the phase-specific limits of agreement. The opposing trends suggest view-specific differences in plane definition or prescription, although phase-dependent CT–MR alignment and multi-heartbeat MR acquisition may also have contributed.

For LAX_3, a shift in the segmentation-derived aortic valve center, potentially influenced by LV outflow-tract blood-pool inclusion, could rotate the plane around the LV long axis and yield a narrower CT cross-section than the manually prescribed MR plane, contributing to the negative bias. Conversely, rotational mismatch between coordinate-defined CT and manually prescribed MR LAX_2 planes could yield a broader CT cross-section and contribute to the positive bias. These geometric explanations remain hypotheses because angular and positional differences between the CT and MR planes were not measured.

Li et al. [2] described an automated approach for LV long-axis localization in CCTA using myocardial outer-contour geometry. In 88 CCTA patients, the computer-derived axis showed good agreement with physician-annotated reference axes, with a median angular error of 4.40°. Their study supports the feasibility of automated LV long-axis localization in single-phase CCTA volumes. The present study extends this prior work to multi-phase cine CCTA by defining a patient-specific cardiac coordinate system and extracting reproducible LAX and SAX slices across the cardiac cycle.

Our validation approach also differs: rather than evaluating agreement between computer-derived axis estimates and expert-defined reference axes alone, we tested whether automated CCTA reorientation and LAX/SAX slice extraction across the cine cardiac cycle could produce LV + LVM area measurements that agreed with cardiac MR. This cross-modality validation provides additional evidence by assessing whether automated CCTA plane reorientation can generate clinically relevant, quantitative slice-specific LV + LVM measurements that are consistent with an independent reference modality in cine imaging.

Other studies also support the development of automated, anatomy-aware cardiac plane reformatting. In cardiac CT, Chen et al. developed a deep-learning approach for blood-pool segmentation and LAX/SAX plane prediction, with validation focused on expert-defined planes and reader variability rather than cross-modality LV + LVM area agreement across cine phases [10]. Fan et al. proposed a self-supervised atlas-prompting framework for automatic view positioning [11]. In cardiac MR, earlier computerized methods supported observer-independent SAX acquisition and cardiac-axis planning [12,13]; learning-based methods have been developed for plane prescription and landmark detection [14,15,16]; landmark-based cardiac view projection has been demonstrated for volumetric 4D flow [17]; and an unsupervised domain-adaptation approach has been proposed to resample axial images toward SAX orientation [18].

In this study, applying phase-15 derived reorientation parameters and slice-plane orientations and positions to all cine phases was a deliberate reproducibility choice intended to mirror the fixed-plane nature of cardiac MR cine prescription. In future work, the framework could update LAX/SAX sampling locations dynamically at each phase by localizing phase-specific anatomic landmarks and repositioning the slice planes accordingly. This dynamic approach may better preserve anatomic correspondence as the ventricle changes shape and position during the cardiac cycle, but its evaluation was beyond the scope of the present study.

Several limitations should be considered. Cardiac MR was used as the reference modality, but it is not a fixed geometric ground truth. During MR acquisition, operator prescription of LAX and SAX planes may depend on localizers, landmark selection, phase selection, basal slice selection, and breath-hold position within the respiratory cycle [3,4]. Prior work has shown that LV long-axis orientation is associated with patient body habitus and may vary across the respiratory and cardiac cycles [19,20]. Acquisition-related spatial mismatch is also possible because MR SAX slices are often acquired across multiple breath-holds, during which inconsistent breath-hold position can cause inter-slice respiratory motion [21]. These factors may lead to inconsistent geometric positioning of the MR LAX and SAX reference slices and affect cross-modality agreement.

Beyond MR acquisition-related variability, the CT-MR comparison required approximate phase matching. Cardiac MR included 30 phases, whereas CCTA included 20 phases; therefore, diastasis, ED, and ES were matched approximately rather than by identical temporal sampling. In addition, heart-rate differences between CT and MR acquisitions, including beta-blocker use before CCTA, may shift diastasis timing and contribute to temporal mismatch.

Because this study used slice-based rather than volume-based matching, small CT-MR differences may also reflect intrinsic plane-matching and slice-selection variability. This design was intentional because the framework was developed to provide reproducible LAX/SAX geometry for future AHA-style regional analysis. For SAX comparison, agreement was assessed at three representative SAX locations rather than across the full SAX stack to avoid the most basal and most apical slices, where LV outflow-tract inclusion, basal motion, and incomplete apical coverage can complicate slice matching.

Segmentation-related variation is another potential source of mismatch because LV + LVM area measurements in the two modalities were derived from modality-specific automated segmentation workflows. Direct angular and positional errors relative to expert CCTA reformats, as well as inter- and intra-reader variability in manual CCTA reorientation, were not assessed. Taken together, some CT-MR differences observed in this study may reflect variability in MR plane prescription and acquisition, patient motion, phase matching, slice selection, or segmentation and therefore cannot be attributed solely to automated CCTA reorientation.

Finally, future work should evaluate larger and more diverse cohorts, include patients with abnormal ventricular morphology, assess CT and MR inter-reader reproducibility, quantify angular and positional differences relative to expert-reader references, compare automated and expert CCTA planes, quantify angular and positional errors and reader variability in manual CCTA reorientation, and evaluate the effect of automated CCTA reorientation on downstream cine CCTA functional analysis.

## 5. Conclusions

The proposed automated framework defines a patient-specific cardiac coordinate system for extracting LAX and SAX slices in cine CCTA. CCTA-derived LV + LVM area measurements showed strong agreement with cardiac MR at diastasis, ED, and ES, with the strongest agreement and smallest bias at ED, and small CT-positive biases overall. These results support automated CCTA volume reorientation and reproducible slice extraction as a foundation for future AHA segment-based regional assessment and quantitative functional analysis of cine CCTA.

## Figures and Tables

**Table 1:** Characteristics of the cohort. HR: heart rate; LVEF: left ventricular ejection fraction; BMI: body mass index.

Patient Characteristic	N = 25	Exam Characteristic	CCTA	Cardiac MR
Gender (male)	16 (64%)	HR (bpm)	59.0 ± 7.2	74.2 ± 13.7
Age (years)	52 ± 12	LVEF (%)	53.6 ± 5.2	60.9 ± 3.6
Height (cm)	169.9 ± 12.0	Beta-blocker	Metoprolol	None
Weight (kg)	81.2 ± 17.9	CT-MR interval (hour)	6.1 ± 5.6	
BMI (kg/m^2^)	28.2 ± 6.0			
Clinical history	Suspected valvular disease; no known cardiovascular disease or arrhythmia			

**Table 2:** Summary of phase-specific CT-MR agreement. Each phase includes 150 paired LV + LVM area measurements. Intraclass correlation coefficients (ICCs) summarize agreement between CT- and MR-derived estimates. Bias is defined as CT minus MR; positive values indicate larger CT estimates. Bland–Altman (BA) 95% limits of agreement (LOAs) summarize the spread of CT-MR differences. Linear mixed-effects (LME) 95% confidence intervals (CIs) describe uncertainty in the estimated mean bias after accounting for repeated measurements.

Phase	ICC (95% CI)	Bias (cm^2^)	BA 95% LOA (cm^2^)	LME 95% CI (cm^2^)	LME *p* Value
Diastasis	0.927 (0.895 to 0.950)	1.05	−8.90 to 11.00	−0.11 to 2.21	*p* = 0.076
End-diastole	0.950 (0.928 to 0.966)	0.47	−8.51 to 9.45	−0.58 to 1.52	*p* = 0.376
End-systole	0.919 (0.887 to 0.943)	1.86	−6.29 to 10.02	0.93 to 2.80	*p* < 0.001

**Table 3:** Descriptive CT-minus-MR bias by cardiac phase and LAX/SAX slice. Values are mean differences in cm^2^. Positive values indicate larger CT estimates; negative values indicate larger MR estimates.

Phase	LAX_2	LAX_3	LAX_4	SAX_B	SAX_M	SAX_A
Diastasis	5.10	−4.59	2.25	2.37	1.35	−0.18
End-diastole	3.35	−4.86	1.79	2.37	1.02	−0.82
End-systole	4.62	−2.16	3.81	3.49	1.20	0.23

## Data Availability

The datasets analyzed in this study are not publicly available because they contain patient imaging data subject to confidentiality and institutional review board restrictions. Requests to access the datasets should be directed to the corresponding author and will be considered subject to applicable approvals and data-use agreements.

## Abbreviations

2D	two-dimensional
3D	three-dimensional
AHA	American Heart Association
CCTA	coronary computed tomography angiography
CI	confidence interval
CNN	convolutional neural network
CT	computed tomography
ED	end-diastole
ES	end-systole
ICC	intraclass correlation coefficient
LAX	long-axis
LME	linear mixed-effects
LOA	limits of agreement
LA	left atrium
LV	left ventricle/left ventricular
LVM	left ventricular myocardium
LV + LVM	combined area of the LV cavity and LV myocardium
MR	magnetic resonance
RV	right ventricle/right ventricular
SAX	short-axis
SCCT	Society of Cardiovascular Computed Tomography
SCMR	Society for Cardiovascular Magnetic Resonance

## References

[1] Cerqueira M.D., Weissman N.J., Dilsizian V., Jacobs A.K., Kaul S., Laskey W.K., Pennell D.J., Rumberger J.A., Ryan T., Verani M.S. (2002). Standardized myocardial segmentation and nomenclature for tomographic imaging of the heart. Circulation.

[2] Li S., Ma T., Guo Y., Xiao Y., Yang A., Xu Y., Li S., He Y. (2026). A computer-aided method for the automatic localization of the left ventricular long axis on coronary computed tomography angiography. Quant. Imaging Med. Surg..

[3] Kramer C.M., Barkhausen J., Bucciarelli-Ducci C., Flamm S.D., Kim R.J., Nagel E. (2020). Standardized cardiovascular magnetic resonance imaging (CMR) protocols: 2020 update. J. Cardiovasc. Magn. Reson..

[4] Schulz-Menger J., Bluemke D.A., Bremerich J., Flamm S.D., Fogel M.A., Friedrich M.G., Kim R.J., von Knobelsdorff-Brenkenhoff F., Kramer C.M., Pennell D.J. (2020). Standardized image interpretation and post-processing in cardiovascular magnetic resonance—2020 update: Society for Cardiovascular Magnetic Resonance (SCMR): Board of Trustees Task Force on Standardized Post-Processing. J. Cardiovasc. Magn. Reson..

[5] Bui V., Hsu L.-Y., Chang L.-C., Sun A.-Y., Tran L., Shanbhag S.M., Zhou W., Mehta N.N., Chen M.Y. (2022). DeepHeartCT: A fully automatic artificial intelligence hybrid framework based on convolutional neural network and multi-atlas segmentation for multi-structure cardiac computed tomography angiography image segmentation. Front. Artif. Intell..

[6] Bui V., Shanbhag S.M., Levine O., Jacobs M., Bandettini W.P., Chang L.-C., Chen M.Y., Hsu L.-Y. (2020). Simultaneous multi-structure segmentation of the heart and peripheral tissues in contrast enhanced cardiac computed tomography angiography. IEEE Access.

[7] Bui V., Hsu L.-Y., Shanbhag S.M., Tran L., Bandettini W.P., Chang L.-C., Chen M.Y. (2020). Improving multi-atlas cardiac structure segmentation of computed tomography angiography: A performance evaluation based on a heterogeneous dataset. Comput. Biol. Med..

[8] Yushkevich P.A., Piven J., Hazlett H.C., Smith R.G., Ho S., Gee J.C., Gerig G. (2006). User-guided 3D active contour segmentation of anatomical structures: Significantly improved efficiency and reliability. NeuroImage.

[9] Leipsic J., Abbara S., Achenbach S., Cury R., Earls J.P., Mancini G.J., Nieman K., Pontone G., Raff G.L. (2014). SCCT guidelines for the interpretation and reporting of coronary CT angiography. J. Cardiovasc. Comput. Tomogr..

[10] Chen Z., Rigolli M., Vigneault D.M., Kligerman S., Hahn L., Narezkina A., Craine A., Lowe K., Contijoch F. (2021). Automated cardiac volume assessment and cardiac long- and short-axis imaging plane prediction from electrocardiogram-gated computed tomography volumes enabled by deep learning. Eur. Heart J. Digit. Health.

[11] Fan X., Wang Y., Zhang Y., Bao M., Jia B., Lu D., Gu Y., Cheng J., Zhu H. (2025). AVP-AP: Self-supervised automatic view positioning in 3D cardiac CT via atlas prompting. IEEE Trans. Med. Imaging.

[12] Lelieveldt B.P.F., van der Geest R.J., Lamb H.J., Kayser H.W.M., Reiber J.H.C. (2001). Automated observer-independent acquisition of cardiac short-axis MR images: A pilot study. Radiology.

[13] Jackson C.E., Robson M.D., Francis J.M., Noble J.A. (2004). Computerised planning of the acquisition of cardiac MR images. Comput. Med. Imaging Graph..

[14] Blansit K., Retson T., Masutani E., Bahrami N., Hsiao A. (2019). Deep learning-based prescription of cardiac MRI planes. Radiol. Artif. Intell..

[15] Wei D., Huang Y., Lu D., Li Y., Zheng Y. (2024). Automatic view plane prescription for cardiac magnetic resonance imaging via supervision by spatial relationship between views. Med. Phys..

[16] Xue H., Artico J., Fontana M., Moon J.C., Davies R.H., Kellman P. (2021). Landmark detection in cardiac MRI by using a convolutional neural network. Radiol. Artif. Intell..

[17] Le M., Lieman-Sifry J., Lau F., Sall S., Hsiao A., Golden D., Cardoso M.J., Arbel T., Carneiro G. (2017). Computationally efficient cardiac views projection using 3D convolutional neural networks. Deep Learning in Medical Image Analysis and Multimodal Learning for Clinical Decision Support.

[18] Koehler S., Hussain T., Blair Z., Huffaker T., Ritzmann F., Tandon A., Pickardt T., Sarikouch S., Latus H., Greil G. (2021). Unsupervised domain adaptation from axial to short-axis multi-slice cardiac MR images by incorporating pretrained task networks. IEEE Trans. Med. Imaging.

[19] Engblom H., Hedstrom E., Palmer J., Wagner G.S., Arheden H. (2004). Determination of the left ventricular long-axis orientation from a single short-axis MR image: Relation to BMI and age. Clin. Physiol. Funct. Imaging.

[20] Foster J.E., Engblom H., Martin T.N., Wagner G.S., Steedman T., Ferrua S., Elliott A.T., Dargie H.J., Groenning B.A. (2005). Determination of left ventricular long-axis orientation using MRI: Changes during the respiratory and cardiac cycles in normal and diseased subjects. Clin. Physiol. Funct. Imaging.

[21] Tarroni G., Oktay O., Sinclair M., Bai W., Schuh A., Suzuki H., de Marvao A., O’Regan D., Cook S., Rueckert D. (2018). A comprehensive approach for learning-based fully automated inter-slice motion correction for short-axis cine cardiac MR image stacks. Medical Image Computing and Computer-Assisted Intervention—MICCAI 2018.

